# Neurosyphilis Presenting as an Atypical Case of Posterior Placoid Chorioretinitis in a Young, HIV-Negative Male

**DOI:** 10.7759/cureus.17274

**Published:** 2021-08-18

**Authors:** Matthew J Bierowski, Rui Wang, Hafiza W Javaid, Neil Amin, Alina L Popa

**Affiliations:** 1 Internal Medicine, Penn State Health Milton S. Hershey Medical Center, Hershey, USA; 2 Ophthalmology, Penn State Health Milton S. Hershey Medical Center, Hershey, USA; 3 Internal Medicine, Wellspan York Hospital, York, USA

**Keywords:** ocular syphilis, penicillin g, syphilis, immunology, chorioretinitis

## Abstract

Ocular syphilis can occur at any time after initial infection and most commonly presents as posterior uveitis or panuveitis, although many other ocular findings have been documented. We present the case of a young, otherwise healthy Caucasian HIV-negative male who presented with acute onset of photopsias, floaters, and a rapidly progressive unilateral scotoma who was originally diagnosed with acute zonal occult outer retinopathy (AZOOR) and started on a high dose prednisone taper. Although his clinical symptoms improved on corticosteroids, he was later switched to Penicillin G treatment when his blood and cerebrospinal fluid (CSF) testing demonstrated syphilis as his underlying diagnosis. Given his ocular findings on the exam and reactive syphilitic testing, he was ultimately diagnosed with acute syphilitic posterior placoid chorioretinitis (ASPPC). Our patient's clinical improvement after a high-dose prednisone trial offers further evidence of an autoimmune component to the pathophysiology of ASPPC.

## Introduction

Although the progression of primary to tertiary syphilis tends to follow a chronological course that can span years, both neurosyphilis and ocular manifestations can occur at almost any point following the initial infection; therefore, ocular disturbances and/or central nervous system findings are not a reliable determinant of one’s infectious duration [1]. This makes the diagnosis of neurosyphilis with ocular disturbances challenging due to the disease’s ability to masquerade itself. Case reports have documented a plethora of abnormal presentations, ranging from disorganized behavior and dementia to visual field disturbances, vertigo, and flashing lights [2,3].

Acute syphilitic posterior placoid chorioretinitis (ASPPC) is a rare manifestation of syphilitic invasion of the posterior uvea that is notable for the unique yellow circular placoid lesion presenting in the macula at the outer retina/retinal pigment epithelium (RPE) [4]. Although the underlying pathophysiology remains unclear, it has been suggested that a patient’s underlying immune status may be related to the degree of severity [5]. Although many patients are initiated on corticosteroids at the time of presentation due to unclear etiology, the clinical course of syphilitic uveitis may be exacerbated by immunosuppression and the literature supports this hypothesis [5-7].

We present the case of a young, otherwise healthy Caucasian HIV-negative male who presented with acute onset of photopsias, floaters, and a rapidly progressive unilateral scotoma and was ultimately diagnosed with ASPPC. To our knowledge, there are no case reports describing an HIV-negative individual with positive cerebrospinal fluid (CSF) serology for syphilis presenting with symptoms and findings consistent with ASPPC with clinical improvement upon initiation of corticosteroids.

## Case presentation

A 23-year-old Caucasian male patient initially presented to an Ophthalmology clinic with a six-day history of photopsias and floaters exacerbated by bright light, as well as a one-day history of a rapidly enlarging scotoma in the right eye, progressing from the patient’s inferior visual field. Although he denied any other associated symptoms, he did recall a faint, pinpoint, a painless rash that started on both hands and spread up the forearms approximately three to four weeks prior to the manifestation of his ocular symptoms; however, the rash disappeared within a few days without any further recurrence. While his past medical history was unremarkable, social history revealed a remote history of six sexual encounters with six individuals of the opposite sex.

**Figure 1:**
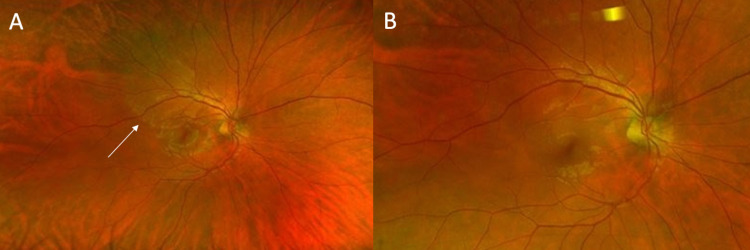
Fundus photo images of the right eye. (A) Cream-yellow lesion present adjacent to the macula (arrow). (B) Resolution of the yellow lesion following penicillin therapy.

**Figure 2:**
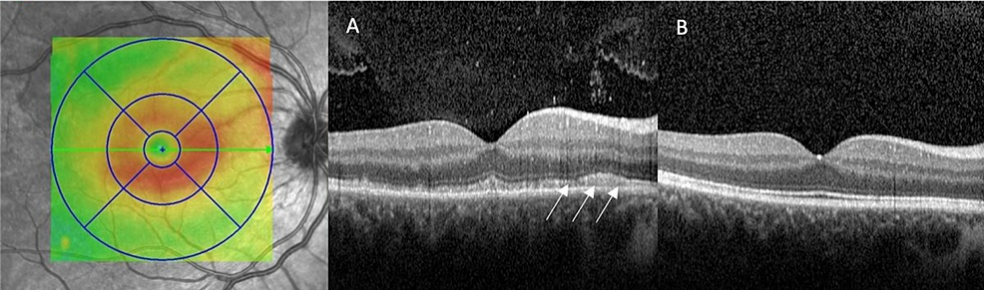
Optical coherence tomography (OCT) of the right eye. (A) Nodular thickening of the outer retinal pigment epithelium with disruption of the inner and outer segment photoreceptor junction (arrows). (B) Reestablishment of photoreceptor junction following penicillin therapy.

**Figure 3:**
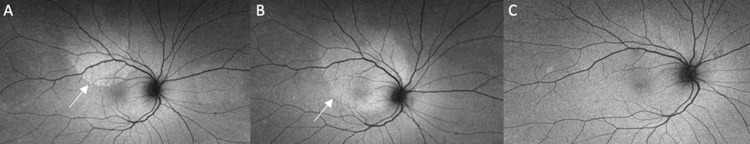
Autofluorescence imaging of the right eye. (A) Superotemporal placoid area of hyperautofluorescence (arrow). (B) Superotemporal placoid area of hyperautofluorescence following two weeks of steroid therapy (arrow). (C) Resolution of the lesion following penicillin therapy.

**Figure 4:**
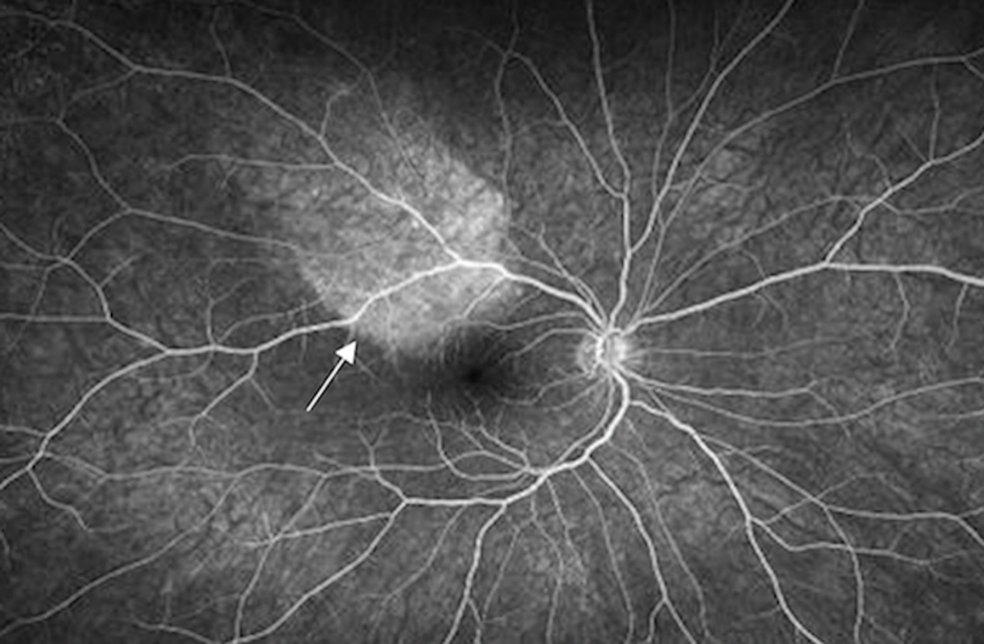
Late phase fluorescein angiography (FA) of right eye demonstrating right superotemporal hyperfluorescent staining with characteristic “leopard spotting” pattern (arrow).

On ophthalmic exam, visual acuity was 20/40 in his right eye (OD) and 20/25+1 in the left eye (OS). Pupils were reactive bilaterally and no relative afferent pupillary defect (RAPD) was appreciated. Confrontational visual field (CVF) testing revealed an inferonasal visual field defect OD, and CVF was full OS. Anterior segment examination was unremarkable bilaterally. Fundus exam was remarkable for a small number of vitreous cells and a cream-yellow placoid lesion superotemporal to the macula OD, which was further appreciated on fundus photography (Figure 1A). Optical coherence tomography (OCT) testing of the macula demonstrated disruption of the inner segment-outer segment (IS-OS) junction line with irregular nodular thickening of the retina pigment epithelium (RPE) and active vitritis (Figure 2A). Fundus autofluorescence (FAF) revealed hyperfluorescence of the superotemporal lesion (Figure 3A) and fluorescein angiography (FA) revealed speckled hyperfluorescence of the superotemporal region during the late phase angiogram (Figure 4).

Although the differential remained broad, given his acute onset of photopsias, floaters, and an enlarging scotoma in conjunction with the CVF and OCT testing results, an initial diagnosis of AZOOR was made. The patient was subsequently started on a high dose steroid taper and both his photopsias and floaters began to improve. Laboratory workup revealed a positive rapid plasma reagin (RPR) titer (1:64), a reactive Treponema pallidum particle agglutination assay (TP-PA), and confirmatory fluorescent treponemal antibody absorption (FTA- AS). HIV, chlamydia, and gonorrhea tests were all negative. A lumbar puncture was also performed revealing clear, colorless CSF with a glucose of 58mg/dL (normal = 40-70mg/dL), protein of 53mg/dL (normal = 15-45mg/dL), four white blood cells (normal = 0-5), no red blood cells, and a reactive 1:1 venereal disease research laboratory (VDRL) test. No pleocytosis was noted. With clear laboratory evidence of syphilitic infection, a characteristic yellow placoid lesion on fundoscopy, and speckled hyperfluorescence during the late phase angiogram, the final diagnosis was presumed to be ASPPC. When his RPR and FTA-ABS serologies returned positive, the prednisone was discontinued and he was referred to an infectious disease specialist for further workup. After CSF studies returned positive for VDRL, he was admitted to the hospital for penicillin desensitization because of a prior amoxicillin allergy. He was then started on a 14-day course of intravenous penicillin G (24 million units/day) via home administration through a peripherally inserted central catheter (PICC) line, as no known resistance of syphilis to penicillin existed. Throughout the course of his antibiotics, the patient continued to describe the improvement of his ocular symptoms. 

At his follow-up visit several months following the initial presentation, the patient’s visual acuity had returned to baseline. CVF demonstrated persistent inferonasal visual field loss of the right eye. However, the fundus photo revealed the resolution of the previously seen yellow outer retinal lesion (Figure 1B), the disrupted right superotemporal photoreceptor junction previously visualized on OCT (Figure 2B), and the placoid hyperautofluorescent lesion on FAF (Figure 3C).

## Discussion

Involvement of the posterior segment is a finding repeatedly seen in late secondary syphilis and is most commonly documented in the literature as chorioretinitis [8], although the spectrum of its involvement is broad and can include vitritis, necrotizing retinitis, retinal vasculitis, and optic neuritis [9]. Three ocular findings have been described in the literature as characteristic of ocular syphilis: “granulomatous” keratic precipitates, iris roseola, and “ground glass” retinitis [10]. While syphilitic testing must be performed if any or all of these findings are present on the exam, they are not always seen as the disease can manifest in almost any part of the eye. Early diagnosis is crucial, as many patients may only present with ocular findings, and the presence of ocular symptoms for greater than 28 days prior to diagnosis has been associated with a worse prognosis [11]. 

ASPPC has been well documented in the literature with Gass et al. first introducing the term in the literature in 1990. In their report, they describe large, yellow-green placoid lesions at the RPE at both the macular and juxtapapillary areas. On FA, early phase angiography demonstrates hypofluorescence, which blends with late-phase hyperfluorescence to create a characteristic “leopard spotting” pattern [4]. Additional imaging findings with OCT include nodular RPE thickening, photoreceptor disruption, as well as punctate hyperfluorescence in the choroid [12].

The pathophysiology of ASPCC remains poorly understood with cases documented in both immunocompetent and immunocompromised subjects. Zamini and Garfinkel suggested that the clinical course of ASPPC is influenced by the patient’s immune status evidenced by their patient developing large placoid macular lesions after starting a regimen of oral prednisone for syphilitic uveitis [5] and this hypothesis is further supported by a case of ASPPC presenting after an intravitreal corticosteroid injection [6]. Furthermore, the clinical course of syphilitic uveitis in HIV-positive patients tends to have a more severe trajectory with relapse possible even after an appropriate course of high dose IV penicillin [7].

The findings, in this case, suggest a differing conclusion, as the patient improved clinically with the administration of systemic steroids prior to being switched to appropriate intravenous penicillin treatment upon return of his positive serology tests. Since syphilitic chorioretinitis is widely considered a form of neurosyphilis, penicillin treatment was required. Nevertheless, the suggestion that the pathogenesis of ASPCC may involve an autoimmune component is supported by our patient noting a substantial improvement in both his floaters and photopsias following steroid therapy. Similarly, both Ormachea et al. and Yoo et al. present cases of ASPPC with negative CSF serology that demonstrated some degree of clinical and physical improvement of the patient’s symptoms after initiating corticosteroids, further supporting a potential autoimmune component to the disease course [13,14]. One proposed mechanism includes an immune-complex mediated hypersensitivity reaction to a treponemal antigen [15] and steroids have been established as the treatment of choice for syphilitic interstitial keratitis, as that pathology is also thought to be secondary to an immunologic phenomenon against treponemal antigens in the cornea [16]. Methotrexate has demonstrated efficacy in reducing inflammation in some forms of syphilitic uveitis as well [17].

With contrasting viewpoints on the underlying mechanism of ASPPC, we do agree that two underlying pathways may both play a role in the formation of ASPPC lesions and their clinical manifestations [13]. Unlike previously reported cases, which demonstrated some degree of improvement of the placoid lesion after initiating immunosuppressants [13,14], our patient's lesion increased in size following steroid therapy (Figure 3B). This finding indicates that a direct spirochete infection, exacerbated by steroid usage, was the dominant pathway in the underlying pathophysiology. This is further supported by our patient having positive CSF serology, unlike previously reported cases, indicating a more advanced infection. Yet, with dramatic improvement in his clinical symptoms after initiating prednisone, it is likely an immune-mediated hypersensitivity component was present.

A low threshold should be present for testing for syphilis in patients with new-onset ocular symptoms. Furthermore, guidelines suggest that in any patient with a positive non-treponemal test and a clinical scenario concerning neurosyphilis, or if the patient demonstrates any otologic or ophthalmologic manifestations, a lumbar puncture should also be performed to test the CSF [18]. While little is known about the relationship between asymptomatic neurosyphilis (ANS) and symptomatic neurosyphilis (SNS) in HIV-negative individuals, prior studies have identified various factors including age, sex, titer levels, and CSF levels that may modify this relationship: development of SNS from ANS was associated with age greater than or equal to 45 years old, male gender, high RPR titers, and high CSF protein concentration [19]. Our patient was fortunate to receive prompt treatment as he possessed three of these four described risk factors (male gender, high RPR titer, and high CSF protein concentration) thus putting him at high risk for progression from ANS to SNS.

## Conclusions

Ocular syphilis should be considered in any patient presenting with abnormal ocular findings and risky sexual behavior. Any patient with positive serologies for syphilis and neurologic, ocular, or otologic manifestations should receive syphilitic CSF testing to not only rule out ANS but also in an effort of risk stratification and ensure appropriate treatment. Although the pathophysiology of ASPPC has yet to be elucidated, the findings in this case report suggest that both an autoimmune component and direct infection by the spirochete may contribute to the clinical course. Further research is necessary to investigate and validate this conclusion.
